# Clinical Performance Comparing Titanium and Titanium–Zirconium or Zirconia Dental Implants: A Systematic Review of Randomized Controlled Trials

**DOI:** 10.3390/dj10050083

**Published:** 2022-05-12

**Authors:** Paulo Rafael Esteves Fernandes, Ada Isis Pelaez Otero, Juliana Campos Hasse Fernandes, Leonardo Mohamad Nassani, Rogerio Moraes Castilho, Gustavo Vicentis de Oliveira Fernandes

**Affiliations:** 1 Faculty of Dental Medicine, Universidade Católica Portuguesa, 3460-525 Viseu, Portugal; paulorafaelfernandes1997@gmail.com (P.R.E.F.); adaisis@yahoo.es (A.I.P.O.); 2 Private Clinic, 3505-606 Viseu, Portugal; juchfernandes@yahoo.com; 3 Division of Restorative and Prosthetic Dentistry, The Ohio State University College of Dentistry, Columbus, OH 43210, USA; nassani.1@osu.edu; 4 Department of Periodontics and Oral Medicine, University of Michigan, Ann Arbor, MI 48109, USA; rcastilh@umich.edu

**Keywords:** zirconia, titanium, dental implants, clinical studies, systematic review

## Abstract

Objectives: This study aimed to compare clinical results between titanium (Ti), zirconia (Zr), or titanium–zirconium (TZ) dental implants and to analyze survival rate (SR), bleeding on probing (BoP), marginal bone loss (MBL), and/or probing depth (PD). Data source: Manual and electronic searches were conducted (PubMed and Web of Science) to identify randomized controlled trials that compared the outcomes of at least two implant types (control and test group) within the same study. The focused question was determined according to the PICOT strategy. Seven studies were included out of 202 research studies initially found. The follow-up periods ranged from 12 to 80 months, and the mean age was from 43.3 to 65.8 years old. The SR for Ti, TZ, and Zr implants ranged from 92.6% to 100%, 95.8% to 100%, and 87.5% to 91.25%, respectively; MBL for Ti, TZ, and Zr implants varied from −1.17 mm to −0.125 mm for Ti, −0.6 mm to −0.32 mm for TZ, and −0.25 mm to −1.38 mm for Zr. Studies showed a low incidence of mucositis and peri-implantitis; however, BoP for Zr was 16.43%, Ti ranged between 10% and 20%, and TZ from 10% to 13.8%. PD for Ti ranged from 1.6 mm to 3.05 mm, TZ was 3.12 mm (only one study), and Zr ranged from 2.21 mm to 2.6 mm. Conclusion: All three types of implants showed similar tissue behavior. However, the TZ group had better results when compared with Ti and Zr for SR, MBL, and BoP, except for PD. Furthermore, the worst SR was found in the Zr implants group.

## 1. Introduction

Over the past 50 years, dental implants have been extensively used in dental treatments to replace missing teeth, accomplishing high predictability and serving as prosthetic treatment support [1,2]. In this context, titanium alloy is considered the gold standard material for fabricating dental implants [1,3]. Thereby, titanium implants (Ti) have had a higher success rate in the long term [2,4], with studies showing survival rates over 95% after 10 years [2,5], confirming its preference (titanium) as the main material [6].

Pursuing to improve the results previously obtained, changes to the implant’s surfaces and composition have been suggested [7]. One of the best surface treatments known is sandblasting of the implant surface with a large-grid and acid-etching technique, which provides moderate roughness and texture, allowing to obtain a better impact at the early phase of osseointegration [8].

Even though Ti implants are biocompatible and have high rates for survival and success, aesthetics is one of the main issues we may encounter, such as discoloration of the peri-implant soft tissue, mainly due to a thin gingival phenotype. Hypersensitivity is also observed due to corrosion of the implant surface [9]. Within this scenario and trying to solve the aforementioned problems, zirconium dioxide (zirconia, ZrO_2_) implants were introduced, presenting a favorable color when compared with Ti implants, combined with biocompatibility and a reduced affinity to bacterial plaque, making this implant a viable choice due to the downsides found in titanium implants [10].

Structure-wise, zirconia dental implants are tetragonal and may be denatured in the presence of water, transforming the tetragonal shape into a monoclinic one, which results in progressive deterioration [11,12]. Therefore, the deterioration might not be clinically relevant, which may not affect all types of zirconia implants. Moreover, there are two setting types for Ti and Zr implants: one- or two-piece implants. The literature reports that the most utilized Zr implants are one-piece [13,14,15], although there is a great availability in the two-piece ZrO_2_ implants, which have some limitations associated with prosthodontic requirements, especially due to their tridimensional position and cementation. However, Zr implants have a high structural strength, are resilient to fracture, and have a greater resistance to corrosion and wear [16]. An in vivo study with different loading designs showed that the bone-to-implant contact (BIC) ranged between 66% and 81% for Zr implants [17]. Rocchietta et al. [16] tested Zr implants in rabbits, with the surface chemically modified, in order to verify and compare the osseointegration values. The authors concluded that they did not find any benefits or statistical significance when comparing the test against the control group studied (Ti implants).

When comparing, we should take into consideration that titanium alloys suffer dissolution and could modify the natural oral microbiome; however, zirconia has a reduced bacterial plaque affinity [17]. Although Zr implants do not have enough background as titanium, their survival rates have been very promising. As mentioned before, Ti implants have a high success rate after 10 years (96.4%) compared with 87% and 100% survival rates shown in recent studies of zirconia dental implants, with follow-up between 1 and 8 years [18].

In addition to these two options (Ti and Zr), another composition for dental implants has been formulated to combine the properties of both titanium and zirconium to develop titanium–zirconium implants (TZ). Nonetheless, these implants did not resolve the aesthetic problems due to still having a metallic appearance; however, they have higher resistance when compared with Ti, thus preventing possible failure due to fracture [19]. Studies have shown that the survival rate of the TZ implants is above 97% after 3 years of functional loading [20,21]. Hence, the proposal of this systematic article was to assess and compare, directly and systematically, the clinical results obtained for Ti and Zr/TZ implants, with clinical parameters as primary outcomes (survival rate, marginal bone loss, probing depth, and bleeding on probing) and, as secondary outcomes, possible interference in the results through analysis of the implant features and patient habits such as smoking.

## 2. Data Sources

This systematic review was conducted following the Preferred Reporting Items for Systematic reviews and Meta-Analysis (PRISMA) guidelines [22], with the focus question determined according to the Population, Intervention, Comparison, Outcome, and Time (PICOT) strategy [23]. The protocol was registered on PROSPERO (CRD42021236781, provided by the Centre for Reviews and Dissemination/CRD—University of York).

### 2.1. Focused Question

The focused question was: “In clinical studies with partially and fully edentulous patients (P), the oral rehabilitations with zirconia or titanium–zirconium implants (I), when compared with titanium (C), did exhibit differences in the clinical outcomes (O), with a follow-up of at least 6 months (T).”

### 2.2. Information Sources and Search Strategy

An electronic search was performed in MEDLINE/PubMed and Web of Science (WoS) with a specific search strategy combining terms (Table 1). An additional manual search evaluated the references of the included articles to verify other possible relevant publications. Only English language articles published in the literature within the last 10 years were included. Two reviewers (P.R.E.F. and G.V.O.F.) developed the electronic and manual search independently. The articles obtained were imported into EndNote X9 (Thomson Reuters, Philadelphia, PA, USA) and subsequently screened.

### 2.3. Inclusion Criteria

This study included randomized and controlled trials (RCTs) with at least 10 patients treated and published in the English language between the period of January 2010 and December 2020; being partially or fully rehabilitated with at least two of the following implants (titanium, zirconia/titanium, or zirconium dental implants); a minimum follow-up of 6 months after functional loading; studies must contain detailed information on the implant placed and report details regarding survival, success, and/or failure rates; only publications with the longest follow-up were included.

### 2.4. Exclusion Criteria

The exclusion criteria were RCTs that enrolled only one type of implant (only titanium, or titanium–zirconium, or zirconia), animal and “in vitro” studies, questionnaires and case series/reports, systematic/critical/narrative reviews, and publications involving patients with ASA Physical Status 3 or more were also excluded.

## 3. Resources Selection

### 3.1. Studies Selection

Duplicated articles were eliminated, and the remaining articles were screened by title and abstracts for eligibility. Articles that did not provide enough information regarding criteria were also included and evaluated. In case of any disagreement, a third author (J.C.H.F.) was introduced, and Cohen’s kappa test was adopted to evaluate the reviewers’ agreement.

### 3.2. Data Extraction and Method of Analysis

The reviewers extracted the data independently for further analysis using data extraction tables (Excel software, Microsoft^®^, v.16), which included the following parameters: author(s), year of publication, mean observation period, the number of patients and implants at the initial stage of the research, mean age of patients, gender, the number of patients and implant dropouts, the number of early and late implant failure, cumulative implant success/survival rate (%), smoking habits, biological complications and peri-implant MBL (mm), setting of the implants and diameter, bleeding on probing (BoP), marginal bone loss (MBL), and probing depth (PD).

### 3.3. Risk of Bias and Quality Assessment

Assessment of the risk of bias and each study’s quality of the included investigations was performed by two reviewers (P.R.E.F. and G.V.O.F.) independently. The Cochrane risk-of-bias tool for randomized trials version 2 (RoB 2) was applied, and the randomization process, groups similar at baseline, blinded group allocation, random housing, blinded interventions, random and blinded outcome assessment, reporting of dropouts, and other biases (funding) domains were addressed. If there was only one red box or two yellow boxes, it was indicative of existent bias for the respective study included. Only if all boxes were green could it be said that no bias was found.

## 4. Review

### 4.1. Study Selection

**Figure 1:**
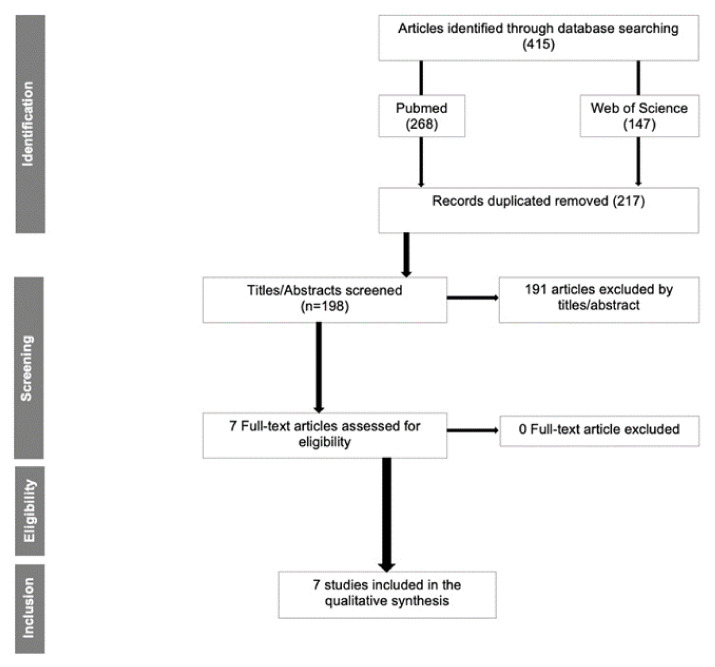
Flow diagram for the search strategy and selection process for included studies.

Initially, 415 articles (268 from PubMed and 147 from Web of Science) were found; of those, 217 duplicated articles were removed. The initial screening was conducted by title and abstract (198 articles). Sequentially, 191 articles were removed due to not meeting the inclusion/exclusion criteria (animal, “in vitro,” interviews, case reports/case series, reviews, and duplicate articles), leaving 7 articles for full-text reading (Figure 1). Cohen’s kappa test was 0.98 and 0.92, respectively, for the first and second steps of the selection.

### 4.2. Risk of Bias

**Figure 2:**
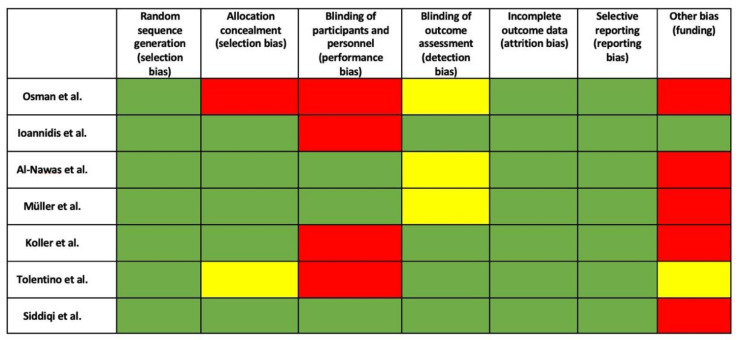
Risk of bias for included RCTs. Low risk (green), unclear risk (yellow), and high risk (red) of bias according to the systematic review.

The risk of bias was evaluated, and bias was observed in all the studies. The highest amount of bias was verified in Osman et al.’s [15] study (three red boxes and one yellow), and the lowest was in Ioannidis et al. [24] and Siddiqi et al.’s [25] studies (only one red box). Al-Navas et al. and Muller et al. presented one red box and one yellow box, Koller et al. had two red boxes, and Tolentino et al. achieved one red box and two yellow boxes. Figure 2 shows the risk of bias for all RCTs included in this systematic review.

### 4.3. Study Characteristics

Within the seven RCT studies enrolled in this systematic review, a total of 260 patients had dental implants placed, and there were 61 total dropouts. Two studies [24,26] did not report how many male and female patients were treated; thus, a total of 71 males and 48 females were reported.

Regarding the number of implants (total number of 646 implants), 169 were Zr, 194 were TZ implants, and 283 were Ti implants. The amount of implant lost during the follow-up period was as follows, 24 Ti (8.48%), 1 TZ (1.03%), and 44 Zr (26.03%). Koller et al. [27] registered 3 out of 31 implants lost (1 Ti out of 15 and 2 Zr out of 16 zirconia implants); Müller et al. [28] registered 2 implants lost (1 TZ and 1 Ti); Ioannidis et al. [24] did not register losses from 40 implants placed (20 for TZ and 20 for Ti); Tolentino et al. [29] also did not observe a loss in 10 implants placed (5 TZ and 5 Ti); Al-Nawas et al.’s study [26] did not appoint the exact number of implants loss, referring that there was this complication. Osman et al. [15] registered a total of 31 implants lost (10 Ti and 21 Zr) out of 129 implants placed (73 Zr and 56 Ti implants), whereas Siddiqi et al. [25] registered 31 losses (10 Ti and 21 Zr) out of 150 implants placed (80 Zr and 70 Ti implants). All data are summarized in Table 2.

### 4.4. Smokers

Each study had a different approach to smoking habits. Koller et al. [27] was the only study that did not refer to any information about the smoking habits of the patients included in the research. Conversely, two studies—Müller et al. [28] and Siddiqi et al. [25]—did not include smokers, although Müller et al. referred to a total of 31 patients without smoking history (66%) and 16 patients who smoked in the past (34%).

Osman et al. [15], Al-Nawas et al. [26], and Tolentino et al. [29] did not include patients who smoked more than 10 cigarettes/day, and Ioannidis et al. [24] included patients who smoked fewer than 20 cigarettes/day. Ioannidis et al. [24] was the only study that compared smokers and nonsmokers, concluding no difference was found between the groups.

### 4.5. Implant Features

Implants were classified according to their diameter and settings (one- or two-piece implant). Two studies [26,29] did not report the type of setting used, three [24,27,28] out of five studies utilized a two-piece implant, and two studies [15,25] used a one-piece implant.

Regarding the diameter, two studies [15,25] reported the utilization of regular and wide-diameter implants for Zr and Ti implants (3.8 mm to 5.0 mm). Three others [26,28,29] placed a narrow diameter (3.3 mm) for Ti and TZ implants. Only one article [24] used a regular diameter for Ti implants (4.1 mm) and a narrow diameter for TZ (3.3 mm), whereas Koller et al. [27] reported regular diameter implants for Zr and Ti (4.1 mm).

### 4.6. Survival Rate (SR)

Regarding the survival rate (SR), the studies with a 1-year follow-up were developed by Tolentino et al. [29], Al-Nawas et al. [26], Osman et al. [15], and Siddiqi et al. [25]. These authors had SR for the Ti implants SR of 100% (5 implants placed), 97.8% (non-reported total), 95.8% (56 implants placed), and 98.57% (70 implants placed), respectively. For the same period for Zr implants, Osman et al. [15] and Siddiqi et al. [25] registered an SR of 90.9% SR (73 implants placed) and 91.25% Zr SR (80 implants placed), respectively. Two studies [26,29] placed TZ implants and obtained an SR of 100% (5 implants) and 98.9% (unknown total).

For the studies with a 3-year follow-up, Ioannidis et al. [24] registered an SR for Ti implants of 97.3% (20 implants placed) and for TZ of 98.7% (20 implants placed). The longest follow-up period analyzed was 6 years and 8 months [27], and the reported SR was 93.33% for Ti implants (15 implants placed) and 87.5% for Zr (16 implants placed). Table 3 summarizes all data found.

For the MBL parameter, all studies presented values that are described in Table 3. For Ti implants, sorted according to decreasing values, Koller et al. [27] found −1.17 mm, Müller et al. [28] −0.61 mm, Tolentino et al. [29] −0.35 mm, Ioannidis et al. [24] and Al-Nawas et al. [26] found −0.31 mm, Osman et al. [15] −0.18 mm, and Siddiqi et al. [25] −0.125 mm.

For TZ implants, also in decreasing order, MBL was recorded by Müller et al. [28] as −0.60 mm, Ioannidis et al. [24] −0.40 mm, Al-Nawas et al. [26] registered a −0.34 mm, and Tolentino et al. [29] −0.32 mm.

Finally, for Zr implants, Koller et al. [27] registered −1.38 mm, Osman et al. [15] −0.42 mm, and Siddiqi et al. [25] −0.25 mm.

### 4.7. Peri-Implant Mucositis and Peri-Implantitis

All clinical data are detailed in Table 3. Regarding mucositis and peri-implantitis, two studies [15,29] did not report or observe these parameters. Three others [26,27,28] did not assess whether mucositis was evaluated. Only two studies [24,25] evaluated the presence of peri-implant mucositis: Ioannidis et al. [24], who had 10 Ti implants and 8 TZ implants affected, and Siddiqi et al. [25], who reported that none of the implants were affected.

Peri-implantitis was evaluated in five studies. Koller et al. [27] had one Ti implant removed due to peri-implantitis, Al-Nawas et al. [26] and Müller et al. [28] had one Ti and one TZ implant with peri-implantitis. Ioannidis et al. [24] had two TZ implants with peri-implantitis, while Siddiqi et al. [25] did not have any implant affected.

### 4.8. Bleeding on Probing (BoP)

BoP was discussed in four out of seven studies, except in Osman et al., Siddiqi et al., and Muller et al. [15,25,28]; it was registered for Ti implants that Koller et al. [27] obtained 12.6%, Ioannidis et al. 20% [24], 10% was reported by Tolentino et al. [29], while Al-Nawas et al. [26] reported a modified BoP, assessing the percentage of patients with BoP (94.5% for Ti and 97.7% for TZ groups) and not the percentage involving the specific implant material.

Koller et al. [27] evaluated BoP for Zr implants and registered 16.43%. Regarding TZ implants, Ioannidis et al. [24] registered 13.8%, and Tolentino et al. [29] 10%. However, Siddiqi et al.’s study [25] stated no significant statistical difference for BoP between Ti and Zr groups.

### 4.9. Probing Depth (PD)

Only three studies reported data for this parameter. Ioannidis et al. [24] registered 2.6 mm for Zr implants and 2.9 mm for Ti, Tolentino et al. [29] reported 3.11 mm for TZ implants and 3.05 mm for Ti implants, and Siddiqi et al. [25] recorded 2.21 mm for Zr and 1.6 mm for Ti implants.

## 5. Discussion

The aim of this systematic study was to verify the clinical data obtained for Zr or TZ implants and compare it against that of Ti implants, verifying the tissue behavior and clinical parameters after loading. This may help to select the best treatments option and build a guide for clinicians. Moreover, TZ implants were considered because it is a mixture of both materials studied, commonly 85% titanium and 15% zirconium, allowing for a direct comparison. In addition, important clinical parameters were considered to develop this review such as MBL, SR, percentage of implants lost, smoking history, mucositis and peri-implantitis, BoP, and PD.

### 5.1. Smoking History

Studies showed that smoking is a risk factor for implant failure and complications [1]. This is an important factor to be analyzed, thus allowing the comparison between smokers and nonsmokers. However, since most studies did not discuss smoking habits, the results of this review did not evaluate the difference between these groups. Nevertheless, it is known that the current literature has suggested that smoking is associated with significantly greater rates of implant failure and marginal bone loss [30].

One study developed by Müller et al. [28] assessed results comparing both groups and concluded that no significant difference was found between them. Another study [27] did not distinguish or evaluate these patients, while only one study did not include smoking patients [25]. Nonetheless, this last study did not specify whether the included individuals were past smokers, which may interfere with the study results.

Three other studies [15,26,29] enrolled smoking patients, considering fewer than 10 cigarettes/day; one study [24] included patients with smoking habits of fewer than 20 cigarettes/day, and this study’s conclusion stated there was no difference between nonsmokers and smokers.

### 5.2. Survival Rate (SR)

Regarding survival rate, titanium implants achieved over 95% SR in 10 years of follow-up [2], which is considered a long-term evaluation. Five out of seven of the studies included in our systematic review surpassed this percentage, but a lower follow-up period was reported. Studies with 1-year follow-up [15,25,26,29] found SR ranging from 95.8% to 100%. After 3 years [24], a value of 97.3% was reported for titanium implants. Nevertheless, the other two studies had a lower SR due to the greater evaluation period, with 92.6% after a 5-year follow-up as reported by Müller et al. [28], and 93.33% after 6 years and 8 months of follow-up as recorded by Koller et al. [27].

For zirconia implants, the SR found in a systematic review [31] was 92% after 1 year of functional loading, which is considered a low percentage compared with that of titanium implants in the same period of time [15,25,26,29]. Similarly, Osman et al. [15], Siddiqi et al. [25], and Koller et al.’s [27] studies found a lower SR for zirconia, with values of 90.9% and 91.25% after 1 year [15], and 87.5% after 6-year and 8 months [25] of follow-up. This fact was corroborated by a meta-analysis [32] that obtained an SR of 91.5% for 1948 zirconium implants, with a mean follow-up of 42.37 months.

When the follow-up period increased, a reduced SR was found for zirconia implants, such as 77.3% after a 7-year follow-up [33]. Although two studies [15,27] did not find a significant statistical difference between titanium and zirconia groups for implants loss, one other [25] concluded that Ti implants had a superior SR than Zr, in line with the literature, which showed a significant difference between them, with a reduction in SR for Zr when compared with Ti implants [32].

Regarding the SR for TZ implants, a meta-analysis showed a mean of 97.7% within 2 years of follow-up [20], similar to values found in the results of our systematic review of between 98.9% [23] and 100% [28] over 1-year follow-up, 98.7% over 3 years [24], and 95.8% over 5 years [28]. These results show that the TZ implants had a closer SR for Ti implants and a higher SR than zirconia.

Our findings show that TZ implants reached a better SR over the years when compared with Zr and Ti implants. However, long-term studies with longer follow-ups are needed to confirm these findings. Nonetheless, the data collected suggested Zr implants had the lowest SR over time compared with the other types of implants (Ti and TZ).

### 5.3. Marginal Bone Loss (MBL)

For MBL of titanium implants, it was possible to verify that the results of the research had great similarities. Koller et al. [27] had the highest MBL with −1.17 mm (after 6 years and 8 months), followed by Müller et al. [28] with −0.6 mm (after 5 years), and Tolentino et al. [29] with −0.35 mm (after 1 year). Al-Nawas et al. [26] and Ioannidis et al. [24], with 1- and 3-years follow-up, respectively, registered equal MBL (−0.31 mm). The studies with the lowest MBL, Osman et al. [15] and Siddiqi et al. [25], had a 1-year follow-up, with values of −0.18 mm and −0.125 mm, respectively. The greatest MBL found was in Koller et al.’s study, which could be because it had the longest period of analysis and because of the type of restoration (lithium disilicate on single implants).

Concerning MBL of Zr implants, the studies showed a range from −0.67 mm [34] to −0.89 mm [32]. Within this systematic review, three studies, Koller et al. [27], Osman et al. [15], and Siddiqi et al. [25], registered MBL of −1.38 mm, −0.42 mm, and −0.25 mm, respectively. These results suggest that the MBL of Ti and Zr implants did not differ over time. However, the studies had a small follow-up period.

A meta-analysis performed by Altuna et al. [35] showed MBL of −0.41 mm after 2 years for TZ implants, whereas Badran et al. [36] registered −0.7 mm. Similarly, the results of our review did not differ significantly from those shown [35,36], whereby four studies [24,26,28,29] found MBL of −0.40 mm, −0.34 mm, −0.60 mm, and −0.32 mm.

Even though the results found for the TZ implants had similar MBL to Ti and Zr implants [35,36], it must be highlighted that there was a low number of TZ implants evaluated, which may be a possible obstacle to drawing any solid conclusion.

### 5.4. Mucositis/Peri-Implantitis

Regarding the biological complications involving peri-implantitis and mucositis, few studies assessed these items. Indeed, only two studies assessed the presence of mucositis. Ioannidis et al. [24] recorded 10 out of 20 Ti implants with mucositis (50%) and 8 TZ implants out of 20 placed (40%), while Siddiqi et al. [25] did not report any case of mucositis out of 80 Zr implants and 70 Ti dental implants. Those results might be an indication that Zr implants have a lower bacterial affinity and, thus, a low prevalence of mucositis [10] when compared with Ti implants. Furthermore, those studies that did not evaluate the presence of mucositis also reported a limitation in the data gathered [15,26,27,28,29].

For peri-implantitis, two studies did not evaluate this parameter [15,29], while the others recorded a very low incidence of peri-implantitis, with only one or two implants affected [24,26,27,28]. Only one study did not have any incidence of peri-implantitis [25]. All data obtained agrees with the data found in the literature, which shows similar results for peri-implantitis involving titanium and zirconia implants [3].

### 5.5. Bleeding on Probing (BoP) and Probing Depth (PD)

When analyzing the result for BoP and PD, only a few studies reported these characteristics. Regarding BoP, it cannot be concluded whether Ti, Zr, or TZ implants had a higher, lower, or similar BoP, as the sample size was too small.

Al-Nawas et al. [26] did not analyze the BoP of the implant sites. Conversely, only one showed the percentage of patients who had general bleeding (94.5% for Ti and 97.7% for TZ groups of patients). Moreover, Siddiqi et al. [25] mentioned a general bleeding index, evaluating the maxilla or mandible but not specifying the implant site bleeding.

After evaluating the PD and due to the limited sample size assessed, it was not possible to obtain a conclusion. However, the PD of Ti, Zr, and TZ implants seemed to be similar, as reported in the literature [3].

### 5.6. Study Limitations

This review presents limitations due to the low number of RCT studies available that analyze Ti and TZ or Zr implants and the risk of bias present in the available literature. Moreover, it was not impossible to perform a meta-analysis because of the different follow-up periods, the fact that only RCT studies were included, and the differences between the inclusion and exclusion criteria of the research included. Furthermore, it was not possible to include TZ implants as a separate group since it is made by a mixture of Ti and Zr and not only by one of those materials.

It should also be considered that of the seven included studies, besides the different follow-ups, four reported 1-year of follow-up data. In addition, this review only focused on the implant materials (i.e., Ti, Zr, and Ti–Zr), and the dental implant design, surface characterization, and prosthetic parameters (such as the type of implant–prosthetic connection, the morphology and material of the abutment, the design and material of the screw, tolerances between screw and thread, the morphology of the implant fixture, the type of prosthetic rehabilitation, and iatrogenic errors) [37] were not considered, which may also influence the clinical performance and result. Finally, the last limitation was the divergent point of analysis and lack of information observed within the included studies.

## 6. Conclusions

Within the limitations of this systematic review and after carefully analyzing all the data, it was possible to conclude that all types of evaluated implants had similar results when analyzing tissue response and behavior, suggesting that Ti, Zr, and TZ implants all allow for a feasible and reliable treatment. However, TZ had a better result when compared with Ti and Zr for SR, MBL, and BoP, and the worst SR was found in the Zr implants group. More randomized controlled clinical studies are needed comparing these types of implants with long-term evaluation and less risk of bias to corroborate the data found in this systematic review. More studies are also needed to evaluate the influence of implant design and surface characterization on clinical performance.

## Figures and Tables

**Table 1:** Search strategy carried out and filters applied.

	MEDLINE (PubMed)	Web of Science (WoS)
#1	P—Edentulous patients treated with dental implants	
	((“Dental Implants” (MeSH Terms)) OR (Dental Implant † (Supplementary Concept)))	‘tooth implantation’ OR ‘tooth implant’ OR ‘dental implant’ OR ‘dental implants’
#2	I—Rehabilitation with zirconia or titanium–zirconium dental implants	
	((“Zirconium” (MeSH Terms)) OR (Zirconium Oxide (Supplementary Concept)) OR (Zirconia (Supplementary Concept)) OR (Yttria Stabilized Tetragonal Zirconia (Supplementary Concept)) OR (“Ceramics” (MeSH Terms)))	‘zirconium oxide’ OR ‘zirconium’ OR ‘ceramics’ OR ‘yttria stabilized tetragonal zirconia’
#3	C—Rehabilitation with titanium dental implants	
#4	O—Clinical outcomes (survival rate, success rate, marginal bone loss, probing in-depth, bleeding on probing, and osseointegration level)	
#5	T—At least 6 months	
Search Combination	(#1 AND #2) No combination conducted done with #3, and #4 since most of the papers on dental implants are about titanium, and the combination with keywords related to outcome would limit the search even more. Item #5 was manually evaluated.	
Filters	English, humans, 10 years	

† is a truncation symbol to retrieve terms with a common root within the database.

**Table 2:** Studies’ characteristics and detailing.

Author	Year	Patients	Period	Mean Age	Gender	Dropout	Titanium Implant (n)	Zirconia Implant (n)	Connection	Company	Implant Lost	Type of Rehabilitation
Koller et al.	2020	22	80 months	46	13M/9F	0	15	16 (Zr)	Morse-taper	Ziterion^®^ (Vario T; Ziterion)	1 Ti/2 Zr	All ceramic (lithium disilicate) single restorations
Müller et al.	2015	47	5 years	72	24M/23F	16	47	47 (TZ)	Morse-taper	Straumann (Bone Level)	1 Ti/1 TZ	Overdentures
Ioannidis et al.	2015	40	3 years	NR	Not clear	NR	20	20 (TZ)	Morse-taper	Straumann	NR	Single-implant crown (porcelain-fused-to-metal crowns) in the anterior or premolar regions
Tolentino et al.	2016	12	1 year	43.3	4M/8F	0	5	5 (TZ)	Morse-taper	Straumann	0	Screw-retained single metal-ceramic crown in the mandible
Osman et al.	2013	24	1 year	62	15M/4F	5	56	73 (Zr)	External hexagon	Southern Implants	10 Ti/21 Zr	Overdentures
Al-Nawas et al.	2011	91	1 year	65.8	NR	21	70	70 (TZ)	Morse-taper	Straumann (Bone Level)	2 Ti/1 TZ	Overdentures
Siddiqi et al.	2015	24	1 year	62	15M/4F	3	70	80 (Zr)	External hexagon	Southern Implants	10 Ti/21 Zr	Overdentures

NR, not reported; M, male; F, female; Ti, titanium; TZ, titanium–zirconium; Zr, zirconia.

**Table 3:** Clinical data obtained from the included studies.

Study	Follow-Up	SR Ti (%)	SR Zr/TZ (%)	MBL Ti (mm)	MBL Zr or TZ (mm)	1 or 2 Pieces	BoP Ti (%)	BoP TZ/Zr (%)	Mucositis	Peri-Implantitis	PD Ti	PD Zr
Koller et al., 2020	80 m	93.3	87.5 (Zr)	−1.17	−1.38 (Zr)	2	12.6	16.4 (Zr)	NR	1 Ti was removed after 80 m	NR	NR
Muller et al., 2015	5 y	92.6	95.8 (TZ)	−0.61	−0.60 (TZ)	2	NR	NR	NR	1 Ti/1 TZ	NR	NR
Ioannidis et al., 2015	3 y	97.3	98.7 (TZ)	−0.31	−0.40 (TZ)	2	20	13.8 (TZ)	10 Ti/8 TZ	2 TZ	2.9 mm	2.6 mm
Tolentino et al., 2015	1 y	100	100 (TZ)	−0.35	−0.32 (TZ)	NR	10	10 (TZ)	NR	NR	3.051 mm	3.1 mm (TZ)
Osman et al., 2013	1 y	95.8	90.9 (Zr)	−0.18	−0.42 (Zr)	1	NR	NR	NR	NR	NR	NR
Al-Nawas et al., 2011	1 y	97.8	98.9 (TZ)	−0.31	−0.34 (ZT)	NR	94.5	97.7 (TZ)	NR	1 Ti/1 TZ	NR	NR
Siddiqi et al., 2013	1 y	98.6	91.2 (Zr)	−0.125	−0.25 (Zr)	1	NR	NR	0	0	1.6 mm	2.2 mm

NR, not reported; SR, survival rate; BoP, bleeding on probing; MBL, marginal bone loss; PD, probing in-depth; Zr, zirconia; TZ, titanium–zirconium; Ti, titanium; m, months; y, year(s).
